# Interaction of Poly L-Lactide and Tungsten Disulfide Nanotubes Studied by In Situ X-ray Scattering during Expansion of PLLA/WS_2_NT Nanocomposite Tubes

**DOI:** 10.3390/polym13111764

**Published:** 2021-05-27

**Authors:** Lison Rocher, Andrew S. Ylitalo, Tiziana Di Luccio, Riccardo Miscioscia, Giovanni De Filippo, Giuseppe Pandolfi, Fulvia Villani, Alla Zak, Gary H. Menary, Alex B. Lennon, Julia A. Kornfield

**Affiliations:** 1 School of Mechanical and Aerospace Engineering, Queen’s University Belfast, Belfast BT9 5AH, UK; l.rocher@qub.ac.uk (L.R.); G.Menary@qub.ac.uk (G.H.M.); a.lennon@qub.ac.uk (A.B.L.); 2 Division of Chemistry and Chemical Engineering, California Institute of Technology, Pasadena, CA 91125, USA; aylitalo@caltech.edu (A.S.Y.); tidilu@caltech.edu (T.D.L.); 3 Division of Sustainable Materials, ENEA, Centro Ricerche Portici, 80055 Portici, Italy; riccardo.miscioscia@enea.it (R.M.); giuseppe.pandolfi@enea.it (G.P.); fulvia.villani@enea.it (F.V.); 4 Division of Photovoltaics and Smart Networks, ENEA, Centro Ricerche Portici, 80055 Portici, Italy; giovanni.defilippo@enea.it; 5 Faculty of Sciences, Holon Institute of Technology, Holon 5810201, Israel; alzak@hit.ac.il

**Keywords:** polylactide, tungsten disulfide nanotubes, nanocomposites, blow molding, WAXS, SAXS, bioresorbable vascular scaffold

## Abstract

In situ synchrotron X-ray scattering was used to reveal the transient microstructure of poly(L-lactide) (PLLA)/tungsten disulfide inorganic nanotubes (WS_2_NTs) nanocomposites. This microstructure is formed during the blow molding process (“tube expansion”) of an extruded polymer tube, an important step in the manufacturing of PLLA-based bioresorbable vascular scaffolds (BVS). A fundamental understanding of how such a microstructure develops during processing is relevant to two unmet needs in PLLA-based BVS: increasing strength to enable thinner devices and improving radiopacity to enable imaging during implantation. Here, we focus on how the flow generated during tube expansion affects the orientation of the WS_2_NTs and the formation of polymer crystals by comparing neat PLLA and nanocomposite tubes under different expansion conditions. Surprisingly, the WS_2_NTs remain oriented along the extrusion direction despite significant strain in the transverse direction while the PLLA crystals (c-axis) form along the circumferential direction of the tube. Although WS_2_NTs promote the nucleation of PLLA crystals in nanocomposite tubes, crystallization proceeds with largely the same orientation as in neat PLLA tubes. We suggest that the reason for the unusual independence of the orientations of the nanotubes and polymer crystals stems from the favorable interaction between PLLA and WS_2_NTs. This favorable interaction leads WS_2_NTs to disperse well in PLLA and strongly orient along the axis of the PLLA tube during extrusion. As a consequence, the nanotubes are aligned orthogonally to the circumferential stretching direction, which appears to decouple the orientations of PLLA crystals and WS_2_NTs.

## 1. Introduction

Dispersing nanoparticles in a polymer matrix can significantly enhance the chemical and physical properties of the polymer. This potential for polymer nanocomposites to outperform neat polymers has motivated decades of growth in academic research and the nanocomposite industry [1,2,3,4]. Bulk performance of a polymer nanocomposite is strongly dependent on the ability of the nanoparticles to disperse homogeneously in the polymer matrix, which is determined by the interaction between the nanoparticle and the polymer [5,6,7,8]. Dispersion remains a key challenge for graphene and carbon nanotubes (CNT) because the van der Waals interaction between nanoparticles leads to agglomerated bundles [9,10]. Functionalization of the nanoparticle surface is a commonly used technique for improving the compatibility between the two-phase material [8,11]; however, in biomedical applications such as scaffolds, functionalization poses the risk of introducing additional material that may be toxic to the body. A better control over desired properties can also be achieved through the manufacturing process [12,13,14] or modification of the microstructure at the interface [15] without adding further components to the composite.

One polymer that may benefit from the addition of nanoparticles is poly-lactic acid (PLA), an eco-friendly, biodegradable thermoplastic. Its biocompatibility, biodegradability (into nontoxic lactic acid), good mechanical properties and processability [16] explain its increasing popularity in a large range of applications, especially in the biomedical field [17,18]. In particular, PLLA (the L-isomer of PLA) is well suited for the production of bioresorbable vascular scaffolds (BVS), which have been developed to heal damaged arteries [19]. While commonly prescribed permanent metal stents remain in the patient for life and can cause late-stage complications, the clinically approved BVS (ABSORB, Abbott Vascular) resorbs into the body within two to three years after implantation [20,21]. The ability of PLLA to biodegrade offers many advantages, including reduced incidence of late stent thrombosis, regained vasomotion function of the artery, and the possibility of reoperation if plaque builds up again [22,23,24]. However, some clinical trials reported inferior outcomes (e.g., lesion failure, vessel myocardial infarction, stent thrombosis) after two to three years for patients with BVS compared to those with conventional drug-eluting stents (DES), which led to the recommendation of continuing the use of conventional DES until BVS performance improves [25]. One reason for the inferior results with BVS is their thicker struts (~150 µm, as compared to ~80 µm for metal stents) [25,26], which disrupt blood flow [27] and slow endothelialization [28]. In this and other applications, the limited strength and brittleness of PLLA in comparison to permanent metal stents also limit its use. To overcome these issues, previous research has investigated the use of alternative copolymers [29,30], the addition of nanoparticles [31,32], and process-induced microstructural modification [33,34].

Flow-induced crystallization (FIC) can enhance the slow crystallization rate of PLA [35] and drive crystal formation to reinforce the polymer. FIC uses shear [36,37,38,39,40,41] or elongational deformation (from melt or glassy state) [33,42,43,44,45] to stretch the polymer chains. This stretching orients the polymer chains and creates precursors for crystal growth along their length. The formation of such precursors (shish) in the flow direction can be followed by the growth of regularly spaced lamellae (kebabs) perpendicular to the shish axis, creating a unique, oriented microstructure [42,46,47]. Studies related to FIC of PLLA nanocomposites observed that high-aspect-ratio nanoparticles (e.g., fibers or nanotubes) tend to align along the flow direction and increase the nucleation density [36,48]. Upon an intense shear flow, “nanohybrid” shish kebabs (NHSK) can form along the nanoparticles when the polymer chains wrap around them well enough to support the epitaxial growth of regularly spaced lamellae. Studies have shown that NHSKs can significantly improve mechanical properties of thermoplastic nanocomposites [40,41,43,45].

Similar to FIC, the blow molding or “tube expansion” stage of BVS manufacturing causes PLLA to undergo dramatic changes in microstructure, including the orientation of the amorphous chains and the formation of oriented crystals [33,34,42,49]. Tube expansion is a key manufacturing step to confer the desired balance of strength and ductility to the BVS scaffold [34] and can significantly affect the properties achieved in later manufacturing steps, especially during the crimping stage before implantation [50]. Because the kinetics of processing affects the microstructural changes, Ramachandran et al. developed a custom-built instrument to probe these modifications during PLLA tube expansion with small- and wide-angle X-ray scattering (SAXS and WAXS, respectively) at a synchrotron X-ray facility [42]. They observed that PLLA expansion occurs in two stages, a first stage of glassy deformation followed by a second stage of rubbery deformation. During the second stage, they observed the growth of a “shish-kebab” microstructure, with shish aligned along the circumferential (θ) direction. They also found that the temperature at which the tube was expanded and the temperature at which the tube was later annealed strongly affected the final microstructure and orientation.

The motivation for the present study on the interaction between PLLA and tungsten disulfide nanotubes (WS_2_NTs) came from the need to improve PLLA mechanical properties for the success of PLLA-based BVS and the recent findings [40,41,43,45] of hybrid nanoparticle-polymer microstructures formed by FIC. WS_2_NTs are a promising choice of nanofiller because of their established synthesis methods [51,52], high aspect ratio, excellent mechanical properties [53], and lack of known cytotoxicity [54,55,56]. Moreover, recent studies on PLA/WS_2_NT nanocomposites blended by melt mixing showed the capacity of WS_2_NTs to easily disperse in the polymer matrix, promote PLA crystallization, and reinforce PLA at low loading (0.5 wt%) [57,58,59,60]. As an additional advantage, WS_2_ could confer enhanced radiopacity to a PLLA-based BVS [61] due to the higher atomic number of tungsten (compared to the carbon and oxygen atoms of the PLLA), which would enable X-ray imaging of the BVS during implantation and follow-up treatment.

In this work, we developed an upgraded version of the rig described by Ramachandran et al. [42] to perform the first study to our knowledge of the impact of WS_2_NTs on the PLLA microstructural changes during tube expansion at temperatures close to the glass transition (T_g_). As in our previous study [42], we recorded the evolution of the strain field during tube expansion with a camera and simultaneously acquired in situ SAXS and WAXS data to compare the microstructures of neat PLLA and PLLA/WS**_2_**NT tubes expanded under varied conditions. We were surprised to find that (1) WS_2_NTs do not reorient significantly despite the large strain applied during expansion of PLLA/WS_2_NT tubes and (2) while WS_2_NTs promote nucleation of PLLA crystals, they do not direct the crystal orientation and do not induce NHSK in PLLA/WS_2_NT tubes.

## 2. Materials and Methods

### 2.1. Synthesis of Tungsten Disulfide Nanotubes (WS_2_NTs)

Inorganic nanotubes of tungsten disulfide (WS_2_NTs) were produced in our laboratory by a vapor–gas–solid (VGS) reaction performed in a specially designed quartz reactor. The main two steps of the reaction are (1) growth of suboxide whiskers (up to 25 µm long and ~100 nm in diameter) from spherical oxide nanoparticles of tungsten trioxide (WO_3_) and (2) sulfurization into WS_2_NTs of the same size. In more detail, during the first step, the nanoparticles precursor is reduced, resulting in the formation of a volatile suboxide phase (WO_2.75_). After undergoing additional partial reduction to WO_2_, the mixed WO_2.75_/WO_2_ vapor promotes condensation and the fast growth of nanowhiskers to a stable suboxide (WO_2.72_) phase. During the second step, the nanowhiskers are gradually converted into WS_2_ nanotubes by a slow, diffusion-controlled sulfurization reaction with H_2_S/H_2_, gases starting from their surface. This reaction proceeds epitaxially from the outside to the inside of the nanowhisker, advancing layer by layer and using the external cylindrical sulfide layers as a template, which ultimately forms a perfectly crystalline and hollow nanotube of WS_2_. Figure S1 in the Supplementary Materials (SM) shows scanning electron microscopy (SEM) and transmission electron microscopy (TEM) images of WS_2_NTs obtained by this process. The two main steps of the reaction, the growth of suboxide whiskers and their sulfurization, occur in the same reactor at elevated temperatures (greater than 800 °C) and under the same H_2_S/H_2_/N_2_ gas flow (N_2_ is a carrier gas), following each other by a self-control mechanism. A detailed description of the reaction route and growth mechanism of WS_2_NTs was reported earlier [51], followed by newer insights [62].

### 2.2. Tube Extrusion

Medical grade PLLA (PL38) was provided by Corbion, Gorinchem, Netherlands. Before extrusion, the polymer was ground into powder using a cryogenic grinder and mixed with the WS**_2_**NTs (0.5 wt%). The material was then dried for 4 h at 120 °C to remove moisture and avoid hydrolytic degradation prior to compounding with a co-rotating 16 mm twin-screw extruder with an L/D ratio of 25 (HAAKE Rheomex OS PTW16, ThermoFisher SCIENTIFIC, Stone, United Kingdom). The temperature of the extruder barrel was kept at 230 °C and the screw speed was fixed at 100 rpm. Neat PLLA tubes were produced by the same process. After extrusion, the tubes were quench cooled to prevent crystallization. The extruded tubes had a nominal inner diameter (ID) of 0.6 mm and an outer diameter (OD) of 1.5 mm.

### 2.3. Tube Expansion Instrument

**Figure 1:**
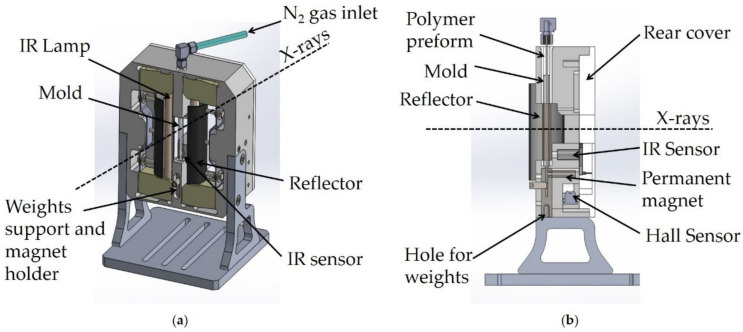
(**a**) A 3D and (**b**) a side view of the portable instrument developed for in-situ scattering experiments during PLLA/WS**_2_**NT tube expansion. The polymer tube is inserted in a Pyrex glass mold mounted vertically on a support and is placed along the X-ray path. Two IR lamps and reflectors facing the mold provide feedback-controlled heating of the polymer tube, while inflation is activated by compressed gas injected from the top (air inlet). The axial elongation of the polymer can be enhanced by application of additional weights and can be measured by a magnetic Hall sensor.

We developed an instrument to control tube expansion while performing X-ray scattering experiments at a synchrotron X-ray source that was inspired by the apparatus presented by Ramachandran et al. [42]. The principle of operation of the system, based on BVS patent found in literature [63], remains the same: a PLLA tubular preform (inner diameter (ID): 0.6 mm, outer diameter (OD): 1.5 mm) is placed inside a Pyrex mold (ID: 3.9 mm, OD: 6.0 mm), where it is heated with IR lamps mounted symmetrically and parallel to the mold and subsequently inflated by compressed nitrogen (N_2_) gas (Figure 1a). In the present design, the tube is mounted vertically (instead of horizontally, as in Ramachandran et al. [42]) to allow the attachment of a small weight to the bottom of the tube to provide a constant load during deformation, which helps with keeping the tube straight and stable during heating (Figure 1b). The vertical movement of the polymer tube is detected through variations in the magnetic field around the weight by a Hall sensor. Additionally, the present rig design uses an IR temperature sensor facing the tube to measure temperature more reliably than a thermocouple and allow for more precise feedback control during heating and activation of the pressure valve at the desired temperature. A thermocouple is placed between the tube and the mold to provide an additional measurement of the temperature. The mechanical assembly is held in place by a platform fixed to the beamline stage that places the tube in the X-ray path (Figure S2).

The present design, like the previous one, uses a process controller to heat the mold and the tube through a linear temperature ramp up to a setpoint temperature. The temperature is stabilized at this setpoint temperature for about 10 min to allow for further expansion and annealing. Additional information on the mechanical assembly and control box can be found in the SM (Figure S3).

### 2.4. In Situ Structural Characterization

Experiments to study microstructural changes during tube expansion with wide- (WAXS) and small-angle X-ray scattering (SAXS) were performed at the Advanced Photon Source, beamline 5-ID-D, at the Argonne National Laboratory (Lemont, IL, USA). The incident X-ray beam is aligned to be perpendicular to the axial direction of the tube and the mold around it (Figure S2). During experiments, 2D scattering patterns are acquired every 0.65 to 1 s with an exposure time of 0.5 s using X-rays with a wavelength of 0.7293 Å. WAXS and SAXS images were acquired on Rayonix CCD detectors with a sample-to-detector distance of 20.05 cm and 8.503 m, respectively. The wave vector q is calibrated using a spinning silicon diffraction grid. We isolate the relatively weaker signal of PLLA tubes (wall thickness of extruded tube ~450 µm and expanded tube ~100 µm) from that of the Pyrex mold (~1 mm thick) in the WAXS patterns by performing the two-parameter background subtraction method described by Ramachandran et al. [42]. Images of the tube, synchronized with the X-ray scattering acquisition, were collected with an optical Guppy camera available at the beamline. These images were used to measure axial elongation and radial expansion.

### 2.5. Expansion Parameters

**Figure 2:**
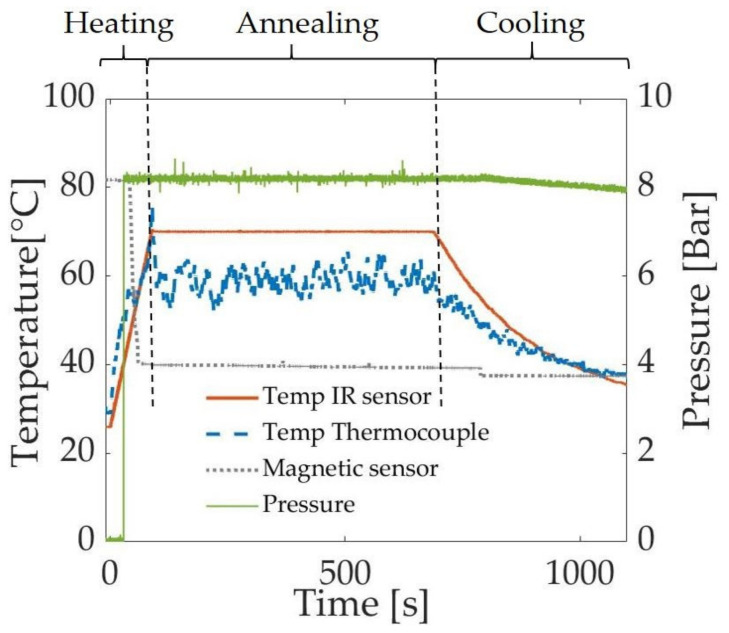
The three steps of the tube expansion protocol: 1—Heating of the tube and opening of the pressure valve at the pressure activation temperature to expand the tube; 2—annealing of the tube at the setpoint temperature for 10 min; 3—passive cooling of the tube to room temperature. This plot comes from the expansion of a neat PLLA tube that was annealed at 70 °C, with 8 bar of nitrogen gas applied when the temperature reached 40 °C.

Tubes were expanded according to a protocol adapted from industrial stretch blow molding processes, with an additional annealing step before cooling to explore further microstructural changes (Figure 2). First, the tube is heated to a “pressure activation temperature” (T_act_), at which the pressure is applied (inside the tube) by opening a valve, causing the tube to expand. After, the tube continues to be heated to the setpoint temperature, at which the expanded tube is annealed for 10 min. Finally, the heating lamps are turned off to allow the tube to cool to room temperature. For each tube expansion, the temperature (as measured by the IR sensor, orange trace in Figure 2, as well as by a thermocouple, blue trace) and the pressure (green trace) are recorded. The signal from the Hall sensor (gray trace, arbitrary units) shows an abrupt decrease when the tube starts elongating.

Three parameters were tested to modify the maximum strain and strain rate during expansion: (1) the annealing temperature (T_ann_), (2) the pressure, and (3) the pressure activation temperature (T_act_). The heating rate was fixed at 30 °C/min for all the expanded samples. All temperatures reported in the results and discussion sections correspond to the temperatures recorded by the IR sensor. While the calibration experiment (described in Figures S4 and S5 of the SI) showed that the temperature measured by the IR sensor could be as much as 20 °C below the temperature in the center of the tube, it was consistent and stable under feedback control. In total, about 20 tubes of neat PLLA and PLLA/WS_2_NT were expanded under different conditions (T_ann_ = {50,60,70,80} °C, T_act_ = {40,50,60} °C, Pressure = {7,8,9} bar). Due to the limited time at the beamline and the complexity of the experiment, we could only perform one successful tube expansion at each set of conditions. Nevertheless, results of experiments performed at close conditions (e.g., same annealing and pressure activation temperatures but different pressure) showed little difference. Before each expansion, a careful visual inspection of the extruded tubes was carried out to exclude tubes with significant defects. Additionally, the tubes presented in this study did not leak upon expansion. While the processing conditions are consistent, the expansion may slightly vary due to small variations of the tube thickness and defects, but we do not expect these to change our conclusions.

### 2.6. Measurement of the Tube Thickness

**Figure 3:**
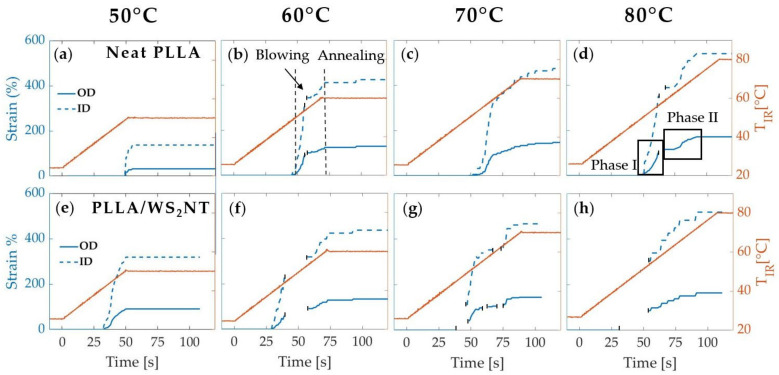
Plot of the strain of the outer diameter (OD, blue solid line) and inner diameter (ID, blue dashed line) and the temperature ramp (orange line) are presented for four different annealing temperatures (50, 60, 70, and 80 °C) for neat PLLA (**a**,**b**,**c**,**d**) and PLLA/WS_2_NT (**e**,**f**,**g**,**h**) tubes. The pressure (7 bar) was activated at 40 °C in all cases. The gaps in the data points indicate time points at which the measurement of the tube diameter (used for strain calculation) was obscured by excess light.

**Figure 4:**
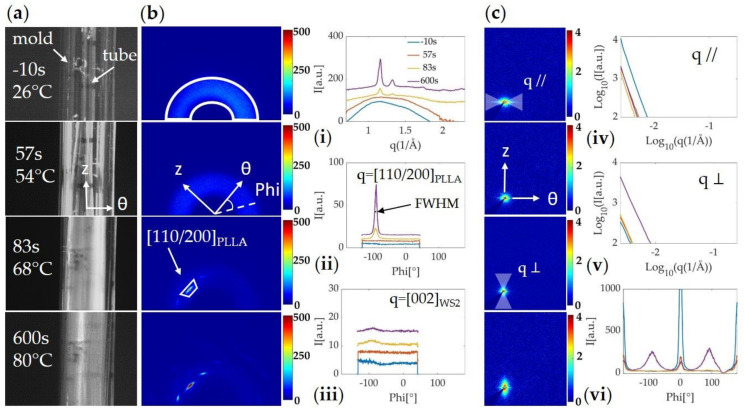
(**a**) Camera images and in situ (**b**) WAXS and (**c**) SAXS patterns acquired during the expansion of a neat PLLA tube pressurized at 40 °C and annealed at 80 °C; (**i**) azimuthally averaged intensity I(q); (**ii**) radially averaged WAXS intensity I(ϕ) integrated around the (110)/(200) of PLLA (from q = 1.12 Å^−1^ to 1.18 Å^−1^); (**iii**) radially averaged WAXS intensity I(ϕ) integrated around the (002) peak of WS_2_ (from q = 0.98 Å^−1^ to 1.04 Å^−1^) for comparison with the PLLA/WS**_2_**NT sample in Figure 5; (**iv**) Log-Log plot of the azimuthally averaged SAXS intensity I(q) in the equatorial direction (mask q//); (**v**) Log-Log plot of the azimuthally averaged SAXS intensity I(q) in the meridional direction (mask q⊥); (**vi**) radially averaged SAXS intensity I(ϕ), integrated over all q values.

**Figure 5:**
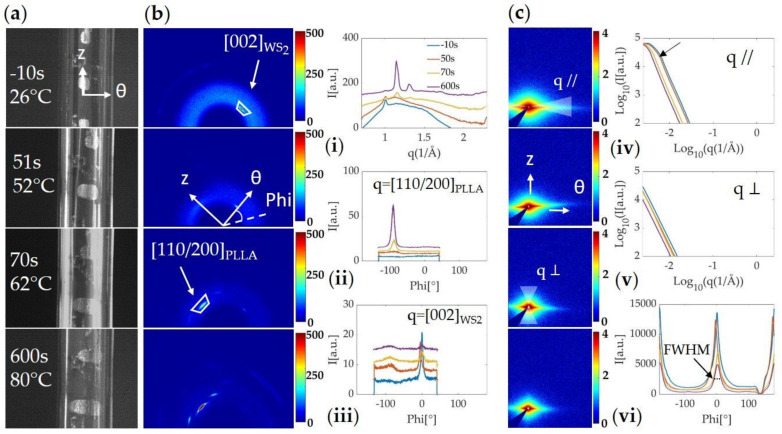
(**a**) Camera images and in situ (**b**) WAXS and (**c**) SAXS patterns acquired during the expansion of a PLLA/WS**_2_**NT tube pressurized at 40 °C and annealed at 80 °C; (**i**) azimuthally averaged intensity I(q); (**ii**) radially averaged WAXS intensity I(ϕ) integrated around the (110)/(200) of PLLA (from q = 1.12 Å^−1^ to 1.18 Å^−1^); (**iii**) radially averaged WAXS intensity I(ϕ) integrated around the (002) peak of WS_2_ (from q = 0.98 Å^−1^ to 1.04 Å^−1^); (**iv**) Log-Log plot of the azimuthally averaged intensity I(q) in the equatorial direction (mask q//), the arrow indicates the “elbow” created by the change of slope; (**v**) Log-Log plot of the azimuthally averaged SAXS intensity I(q) in the meridional direction (mask q⊥); (**vi**) radially averaged SAXS intensity I(ϕ), integrated over all q values.

**Figure 6:**
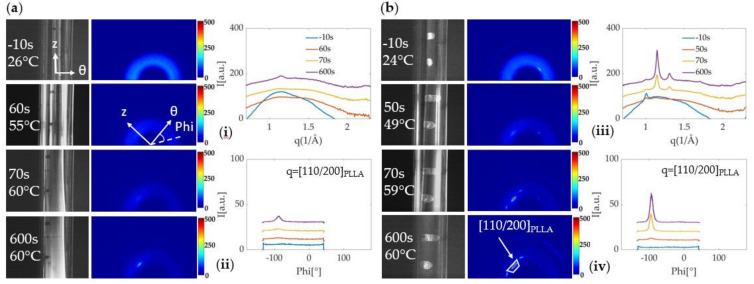
Camera images and in situ WAXS data acquired from tubes annealed at 60 °C (pressure activated at 40 °C). (**a**) neat PLLA with (**i**) Azimuthally averaged intensity I(q); (**ii**) radially averaged WAXS intensity I(ϕ) integrated around the (110)/(200) of PLLA (from q = 1.12 Å^−1^ to 1.18 Å^−1^); and (**b**) PLLA/WS**_2_**NT with (**iii**) Azimuthally averaged intensity I(q); (**iv**) radially averaged WAXS intensity I(ϕ) integrated around the (110)/(200) of PLLA (from q = 1.12 Å^−1^ to 1.18 Å^−1^).

**Figure 7:**
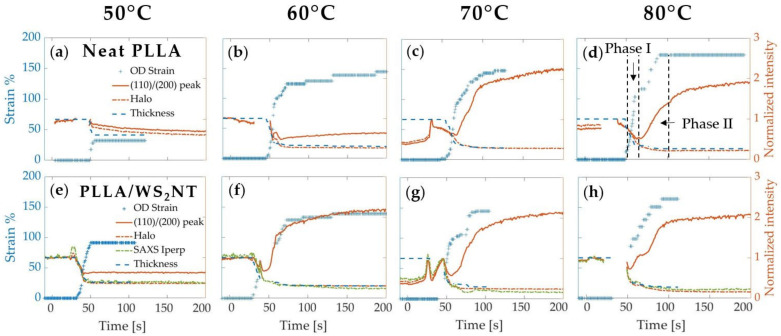
Comparison between deformation and crystallization during tube expansion of PLLA (**a**,**b**,**c**,**d**) and PLLA/WS_2_NT (**e**,**f**,**g**,**h**) at T_ann_ = {50, 60, 70,80} °C showing: the strain (left axis, blue crosses) and the normalized thickness of the tube (blue dashed line), the amorphous halo (dot-dashed orange line) and intensity of the PLLA (110)/(200) peak (orange line). For PLLA/WS_2_NT tubes, another estimate for the normalized thickness is given by the intensity integrated along the meridional direction of the SAXS intensity pattern, labeled as “SAXS Iperp”. Gaps in the OD data indicate that excess light obscured measurement of the tube OD from the camera images; gaps in the X-ray data occur when the tube moves laterally prior to expanding (Figure S6). Note that the reference value corresponds to a moment when the tube was well centered with respect to the incoming X-ray beam. For neat PLLA at T_ann_ = 50 °C, deformation ceased at OD strain of 30% allowing the tube to be off center.

Camera images were used to measure the axial elongation and outer diameter of the tube during experiments. From these measurements, we could then calculate the strain, strain rate, and tube thickness probed by X-rays (equivalent to two times the wall thickness). The radial strain (change in tube diameter) and strain rate, reported in Figures 3 and 7, Figures S7 and S9, were calculated from measurements of the outer diameter (OD) and inner diameter (ID) of the tube using Equations (1) and (2). While the OD was directly measured from camera images, the ID was calculated according to Equation (3), which can be derived by assuming a negligible axial strain (<10% based on the change in marker spacing visible in Figures 4–6) and incompressible tube material.

(1) $$OD\left(ID\right)Strain=\frac{OD\left(ID\right)-OD_{initial}(ID_{initial})}{OD_{initial}(ID_{initial})}$$

(2) $$OD\;strain\;rate=\frac{OD\left(i\right)-OD\left(i-1\right)}{OD_{initial}*\left(time\left(i\right)-time\left(i-1\right)\right)}$$

(3) $$ID=\sqrt{OD^{2}-\left(OD_{initial}^{2}-ID_{initial}^{2}\right)}$$

(4) probed thickness=OD−ID

The thickness of the tube probed by X-rays, reported in Figure 7, is calculated using Equation (4) assuming that the tube is centered in the mold. While this assumption holds during most of the experiment, the tube sometimes shifted from its initial position at the beginning of blowing due to the effects of heat and pressure. Consequently, the actual thickness probed by the X-rays may slightly differ from the result of equation 4 and modify the intensity collected (example given in Figure S6). Because of this uncertainty, we also estimated the thickness by computing the decrease in the WAXS intensity of the amorphous halo as well as the SAXS intensity along the meridional direction (see Discussion).

## 3. Results

Before examining the microstructure, we present the strain during tube expansion (Equation (1)) calculated from the measured OD and the inferred ID (Equation (3)). Here, we consider tubes that were expanded under an imposed pressure of 7 bar that is activated when the temperature reaches T_act_ = 40 °C for four selected annealing temperatures (Figure 3). The annealing temperatures T_ann_, as measured by the IR sensor (and converted to tube temperature based on the calibration in Figure S4), range from 50 °C (~55 °C in the tube), the lowest temperature that permitted deformation, to 80 °C (~100 °C in the tube), at which quiescent crystallization becomes significant for PLLA [64]. When the pressure is activated at 40 °C, the tube expansion is slow enough to be observed over a number of frames (frame rate ~1 s^−1^ and strain rate ~0.04 to 0.01 s^−1^). When T_act_ = 50 and 60 °C (data presented in the SM Figures S7 and S8), the strain rates were higher, and the expansion sometimes occurred over only 1 to 2 frames (strain rate ~1 s^−1^). A pressure of 7 bar was selected to obtain a complete expansion at the highest temperature without causing the tube to burst (often observed at 9 bar). Additionally, variations in the pressure within this range of 7–9 bar had a smaller effect on the strain than did the annealing temperature over the range explored (Figure S9). The axial strain, as measured by the change in marker spacing recorded in the camera images, is less than 10% for all expansions.

The final strain reached by the tube upon expansion monotonically increases with the annealing temperature. At T_ann_ = 50 °C (Figure 3), the neat PLLA tube blows until 30% OD strain, whereas the nanocomposite tube deforms until 90% OD strain. For 60 °C, 70 °C, and 80 °C, both materials reach approximately the same maximum OD strain of 130%, 145%, and 160%, respectively. Moreover, for annealing temperatures higher than 50 °C, two distinct phases can be identified: first, in phase I, the deformation increases until ~90% OD strain (which corresponds to T_IR_ ~50 °C for PLLA/WS**_2_**NT and T_IR_ ~58 °C for neat PLLA); then, in phase II, the strain rate slows down (change of slope) until the end of deformation (Figure 3, upper-right). Compared to the PLLA/WS**_2_**NT tube, the neat PLLA starts its expansion about 15 s later (t ~50 s, T_IR_ ~49 °C vs. t ~35 s, T_IR_ ~42 °C) and a similar offset is observed for the change of slope (t ~65 s, T_IR_ ~58 °C vs. t ~55 s, T_IR_ ~54 °C).

The images used to evaluate strain (e.g., Figure 4a and Figure 5a) are acquired simultaneously with the WAXS (Figure 4b and Figure 5b) and SAXS (Figure 4c and Figure 5c) patterns. The two-dimensional patterns are then integrated to extract 1D profiles: averaging over the azimuthal angle ϕ gives a circularly averaged intensity as a function of q (Figure 4b,i and Figure 5b,i), as well as a selection of 1D WAXS (Figure 4b,i–iii and Figure 5b,i–iii) and SAXS (Figure 4c,iv–vi and Figure 5c,iv–vi) intensity profiles integrated along the azimuthal (I vs q) and radial directions (I vs ϕ) at four different time points during the experiment. These time points capture the polymer tubes before applying heat (t = −10 s, T_IR_ = 26 °C), at the beginning of blowing (t = 57 s, T_IR_ = 54 °C), at the end of blowing (t = 83 s, T_IR_ = 68 °C), and at the end of annealing (t =600 s, T_IR_ = 80 °C). To help with the interpretation, the axial (z) and circumferential (θ) directions of the tubes are indicated by perpendicular white arrows on the 2D X-ray patterns. These directions are given in real space while the 2D X-ray patterns correspond to reciprocal space. For example, PLLA crystals aligned parallel to the z-direction in real space would give a signal along the $q_{∥}$ -direction in the reciprocal space pictured in the 2D X-ray patterns because their periodicity occurs along the θ-direction. Likewise, crystals aligned parallel to the θ-direction in real space would give an intensity along the $q_{⊥}$ -direction in reciprocal space because their periodicity occurs along the z-direction.

At the beginning of the experiment, before applying heat (t = −10 s), the WAXS diffraction pattern shows a strong amorphous halo (area enclosed in the white contour in the WAXS pattern, Figure 4b-left), indicating low crystallinity in the extruded tube. In the SAXS pattern, a streak momentarily appears along the equatorial direction as the tube is moving before expansion (Figure 4c,iv). Based on the camera images, these intense streaks appear when the X-ray beam passes through the outer or inner wall of the tube (Figure S10). Phase I of the expansion (Figure 3-upper right) leads to the thinning of the tube and is associated with a decrease in the amorphous halo intensity (Figure 4b,i). The start of the second phase of expansion comes with the emergence of the θ-oriented (110)/(200) PLLA peak, which rapidly intensifies as the strain increases and slightly continues during the annealing step (Figure 4b,ii). The peak’s full width at half maximum (FWHM), which measures the range of microstructure orientations, indicates a stronger orientation of the PLLA crystals as the tube is expanding. From the time that the tube OD reaches ~110% of strain until the end of the expansion, the SAXS pattern becomes more intense in the meridional direction (purple line in Figure 4c; interpretation given in the SI supported by optical and SEM micrographs in Figure S11).

As a comparison to the tube expansion of neat PLLA shown in Figure 4, we now describe data collected during the blowing of a PLLA/WS_2_NT tube, expanded under the same conditions in Figure 5. At the beginning of the experiment (t = −10 s, Figure 5b), in addition to the amorphous halo, the Bragg peak (002), corresponding to the interlayer spacing in WS_2_NTs, is observed (q = 1.016 Å^−1^) [65]. The azimuthal position of this peak indicates a strong orientation of WS**_2_**NTs parallel to the z-direction of the tube (blue trace in Figure 5iii). This orientation is further evidenced in the SAXS diffraction pattern, where the strong intensity distributed along the equator suggests the presence of well-ordered structures in the extrusion direction (Figure 5vi). In the 1D equatorial profile (Figure 5iv), an abrupt change in slope, which we refer to as an “elbow”, is observed (indicated by the arrow). During the first step of the expansion, the thinning of the tube causes a decrease in the amorphous halo and the Bragg peak of WS_2_ (WAXS) and in the 2D SAXS intensity. As in the expansion of the neat PLLA tube, phase II of the expansion comes with the emergence of the (110)/(200) peak of PLLA parallel to the θ-direction of the tube. In the azimuthal plot of the WS_2_ (002) peak, a faint increase of intensity at ϕ ~(−90°) appears (also present for neat PLLA in Figure 4iii), while the intensity at ϕ ~0° (parallel to the z-direction of the tube) decreases but remains in the same orientation (Figure 5iii). In SAXS, the intensity is localized mostly along the equatorial direction, but the pattern changes from an eye-shaped to a diamond-shaped contour during deformation (Figure 5c-left, top to bottom). From the 1D intensity profile integrated over the equatorial region (Figure 5iv), we can see a shift in the elbow toward lower q-values as the tube becomes thinner.

While an increase of the (110)/(200) peak measured with WAXS was observed during the expansion of both neat PLLA and PLLA/WS**_2_**NT tubes at T_ann_ = 80 °C (Figure 4 and Figure 5), it was qualitatively different when the tube was annealed at 60 °C (Figure 6). Although both neat PLLA and PLLA/WS_2_NT tubes expanded by ~130% (Figure 3), the intensity corresponding to the PLLA (110)/(200) reflection for the PLLA/WS**_2_**NT tube is higher than for neat PLLA (Figure 6b,i) and shows a strong orientation (FWHM ~10 °) in the θ-direction (abrupt appearance of a pronounced peak at ϕ ~(−90 °) between 50 and 70 s in Figure 6b,ii and Figure S12-left). On the other hand, for neat PLLA, the peak in the θ-direction is clearly less intense and broader (FWHM ~16 °) with respect to the nanocomposite tube (Figure 6a,ii and Figure S12-left). To compare the PLLA peak intensity among the different annealing temperatures, Figure S12 in the SI shows the evolution of the intensity from the (110)/(200) peak in the θ-direction (corresponding to the masks shown in Figure 4 and Figure 5) and the corresponding azimuthal width (FWHM) during the expansion and the annealing steps. This figure emphasizes the difference in peak intensity between neat PLLA and PLLA/WS**_2_**NT tubes annealed at 60 °C (Figure S12-left) and shows that this phenomenon is no longer visible for tubes annealed at 70 °C and 80 °C (Figure S12-middle and right), where both neat and nanocomposite samples show a strong increase in the PLLA (110)/(200) peak intensity in the θ-direction during phase II of expansion, which slowly continues during annealing.

## 4. Discussion

In the context of nanocomposites of multiwall nanotubes in polymers, the pair of PLLA and WS_2_NTs has a particularly favorable interaction. The intimate interaction between them is manifested in the nucleation of PLLA crystals by WS_2_NTs. It also causes WS_2_NTs to disperse in PLLA remarkably well because the WS_2_NTs do not aggregate during extrusion. Nevertheless, we believe that the beautiful dispersion of WS_2_NTs in PLLA that results from their intimate interaction also plays an essential role in two remarkable examples of the mutual indifference of nanotubes and polymer: the lack of reorientation of WS_2_NTs during the tube-expansion deformation and the absence of nanohybrid shish kebab morphology around the nanotubes. Below, we discuss reasons for these behaviors and suggest how the good interaction of WS_2_NTs in PLLA could lead to them.

During tube expansion, there are strong correlations among PLLA crystallization, tube expansion, and tube-wall thinning (Figure 7), so measuring them reliably is important. PLLA crystallization is measured by integrating the intensity of the (110)/(200) PLLA peak (integration areas are shown by the (110/200) PLLA peak masks in the 2D WAXS patterns of Figure 4b and Figure 5b). Tube expansion is quantified by the circumferential strain at the outer surface, which is calculated using the outer diameter (OD) observed in the video images. Tube-wall thickness is estimated assuming incompressibility and neglecting axial strain (Equations (3) and (4)) and is normalized by the initial value (Figure 7, blue dashed curve). To justify these assumptions, we compare this estimation of tube thickness to two other quantities that are proportional to tube thickness. The first is the “amorphous halo,” a measure of the intensity of scattering from amorphous PLLA, which is integrated across the mask in the topmost 2D WAXS pattern in Figure 4b and normalized by its value at a reference time shortly before deformation begins. Before detectable crystallization, the normalized amorphous halo closely matches the normalized tube thickness (dashed orange and blue curves decrease together before the solid orange curve increases, Figure 7), validating our estimation of tube thickness up to this point. After the normalized thickness stops decreasing, crystallization sometimes causes a small additional decrease in the amorphous halo intensity, so the amorphous halo may no longer be an accurate metric for the thickness. In the nanocomposites, the strong SAXS signal due to the interface between WS_2_NTs and PLLA enables a further test of the estimate of the normalized thickness: the nanocomposite’s integrated meridional SAXS intensity (integrated across the mask in the 2D pattern of Figure 5c, third row), which we call “SAXS Iperp”, (green dashed curve, Figure 7, bottom row). SAXS Iperp is not affected by crystallization, so it is a reliable metric for tube thickness throughout tube expansion. Indeed, the normalized SAXS Iperp closely matches the normalized tube thickness in Figure 7, providing further confidence in the strain and thickness computed from the video images.

Examining the PLLA crystallization, tube expansion, and tube-wall thinning shows two effects of WS_2_NTs on PLLA: they increase deformation at 50 °C and have a pronounced nucleating effect at 60 °C. At 50 °C, in contrast to the neat PLLA tube (final OD strain ~30%), the PLLA/WS**_2_**NT tube begins to deform 10 s earlier and reaches an OD strain ~90% (Figure 3 and Figure 7, 50 °C, PLLA/WS**_2_**NT). We consider three possible reasons that might give rise to this difference: degradation of molecular weight (Mw) of PLLA catalyzed by WS_2_, greater infrared (IR) absorption by WS_2_NTs, and a plasticizing effect of WS_2_NTs on PLLA. The effect of Mw was ruled out by a study that found that the decrease in Mw after extrusion and drying was small (ca. 15%) and comparable for neat PLLA and PLLA with 0.5 wt% WS_2_NTs. The possible effect of WS_2_NTs on increasing IR heating was confirmed: the PLLA/WS**_2_**NT tubes heated to a temperature 2–3 °C higher than neat PLLA tubes upon IR heating to 60 °C (Figure S5). To isolate the plasticizing effect of WS_2_NTs on PLLA, we performed tube expansion in a heated water bath, without IR exposure. Indeed, the PLLA/WS**_2_**NT tube expanded more than the neat PLLA (Figure S13), showing that WS**_2_**NTs have some plasticizing effect on PLLA. Thus, the effects of WS**_2_**NTs on increasing both IR absorption and mobility of PLLA chains in the glassy state contribute to the interesting effect of WS**_2_**NTs on tube expansion around 50 °C. Increasing T_ann_ to 60 °C modestly increases the crystallization of neat PLLA (*cf*. 50 and 60 °C, neat PLLA, Figure 7 solid orange curves), while crystallization in the nanocomposite increases sharply (*cf*. neat PLLA to PLLA/WS**_2_**NT, Figure 7, second column, solid orange curves). This difference in crystallization clearly reveals that WS_2_NTs promote the nucleation of PLLA crystals, as reported in both quiescent [57] and flow-induced crystallizations [60]. When T_ann_ is increased from 60 °C to 70 and 80 °C (corresponding to an increase in the tube temperature from ~70 °C to ~85 and 100 °C based on the calibration curves in Figure S4), the rate of neat PLLA crystallization increases by an order of magnitude [64]. We suggest that the much faster crystallization of PLLA at these higher temperatures overwhelms the additional contribution to nucleation by the WS**_2_**NTs, such that the effect of WS**_2_**NTs on nucleation is only distinguishable at T_ann_ = 60 °C.

Increasing T_ann_ > T_g_ (T_g_ ~60 °C) also reveals an interplay between deformation and crystallization. As reported previously in neat PLA, the expansion occurs in two steps (blue symbols on Figure 7 at 70 and 80 °C), which is attributed to the transition from a glassy to a rubbery behavior of the polymer chains [42]. In the glassy step, limited segmental rearrangement limits crystallization and, indeed, we observed that the PLLA remains amorphous during phase I of expansion (e.g., the solid orange curve decreases during Phase I in Figure 7, neat PLLA, T_ann_ = 80 °C). In the rubbery step, greater conformational rearrangement of PLLA chains allows crystallization to occur (solid orange curves increase during Phase II in Figure 7) and the segmental preferential orientation in the circumferential direction favors crystals with c-axis in that direction.

**Figure 8:**
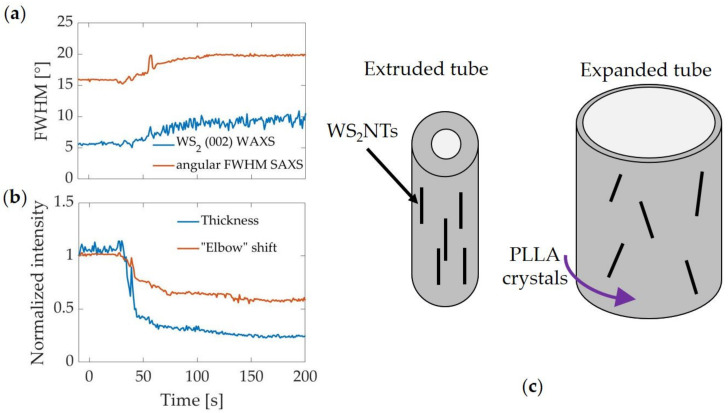
Evolution during the expansion of: (**a**) FWHM of the WS_2_ (002) peak from the WAXS pattern (I vs ϕ) in Figure 5iii and FWHM of I vs ϕ in Figure 5vi; (**b**) tube thickness, normalized by the initial thickness, and the q-value of the “elbow” observed in the 1D intensity in Figure 5iv, normalized by the initial q-value; (**c**) schematic diagram depicting how the WS_2_NTs are initially oriented along the extrusion direction and become slightly less oriented after expansion. The orientation of the PLLA crystals in the circumferential direction is also indicated.

In addition to the effect of WS_2_NTs on the nucleation of PLLA crystals and the deformation behavior near T_g_, we believe that the strong orientation of WS_2_NTs in the extruded tube is also a consequence of the favorable interaction between PLLA and WS_2_NTs. The strength of the alignment of WS_2_NTs in the extruded tube can be seen in the narrow orientation distribution of the WS_2_ (002) diffraction peak (5° FWHM, Figure 8a and Figure S8c, initial value; unlike the ring observed in WAXS patterns of randomly oriented WS_2_NTs in PLA [60]) and the sharpness of the horizontal streak in PLLA/WS_2_NT’s initial 2D SAXS pattern (Figure 5c, t = −10 s; Figure 8a and Figure S8d, initial value). This high degree of alignment is reminiscent of a nanocomposite drawn fiber [66,67] and is unusual for a history of shear flow, where nanotubes that have a higher affinity for each other than for the polymer matrix (like CNT) tend to agglomerate and tumble [9,10]. However, if the particles have a high aspect ratio and behave as individual particles, as is expected for WS_2_NTs based on evidence of their good dispersion in PLA [57,59,60], alignment is expected in shear flow [68,69]. The remarkable alignment of WS_2_NTs in the extruded tube sets the stage for the lack of reorientation observed during tube expansion.

The evolution of the microstructure during circumferential elongation revealed that, despite their close interactions, the nanotubes and polymer chains behave independently in two important ways. The first is that the orientation of the nanotubes is almost unaffected by tube expansion. The orientation of the WS**_2_**NTs during tube expansion broadens only slightly (FWHM of the WS_2_ (002) peak in Figure 5iii broadens from 5° to 9°, blue curve, Figure 8a and Figure S8c; angular distribution of SAXS in Figure 5vi broadens from 16° to 20°, orange curve, Figure 8a and Figure S8c). Additional evidence that there is only mild reorientation of the WS**_2_**NTs is seen in the shift of the q-value of the “elbow” in the SAXS intensity integrated along the equatorial region (arrow in Figure 5iv). This abrupt change in slope marks the transition from the Guinier plateau to the Porod scattering. The scattered intensity becomes independent of q at low enough q (large enough length scales) to encompass the entire scattering entity (Guinier plateau)—the nanotube diameter in the case of equatorial scattering. At higher q, the SAXS intensity decreases as a high power of q due to the sharp interfaces between WS**_2_**NTs and PLLA (Porod). Reorientation causes the “apparent” diameter of the nanotubes to increase, as depicted in Figure 8c, and to shift the SAXS elbow (Figure 8b) to lower q_//_ (higher length scale), which is consistent with the increase in FWHM of the orientation distribution. The low degree of reorientation despite the high stretch ratio (e.g., Figure 3, ID strain >400% for T_ann_ = 80 °C) is surprising. We hypothesize that extrusion aligned WS_2_NTs along the axis of the tube to such a degree that they have almost no component along the circumferential direction to be stretched during tube expansion, so reorientation is small (see the schematic in Figure 8c). As a result, the nanotube orientation is nearly independent of the deformation of PLLA.

The second striking way in which the polymer chains and nanotubes behave independently despite favorable interaction relates to the orientation of the PLLA crystals that form during tube expansion. Based on the literature on nanohybrid shish kebabs (NHSK) [40,41,43,45] and the observation that WS_2_NTs act as a nucleant for PLLA, we expected to see lamellae grow radially outward from the oriented nanotubes. Instead, despite promoting crystal nucleation, WS**_2_**NTs do not appear to direct the orientation of crystallization. If NHSK were present, we would observe a PLLA (110)/(200) peak oriented like the WS_2_ (002) peak in the WAXS pattern because the PLLA chains of kebabs would be aligned with c-axis along the nanotube axis. However, we see no sign of such a structure (Figure 5 and Figure 6b). We consider two possible explanations for the absence of NHSK: proximity to T_g_ and mutual orthogonality. Prior studies that report NHSK in FIC examined conditions in which the history is erased by first heating to the melt, then cooling to a desired temperature for shear or elongation [40,41,43,45]. In the melt, chains rapidly change conformation and, with sufficient subcooling, can rapidly add to a growing lamella as it propagates outward from a nanotube. Motivated by the production process for bioresorbable scaffolds [63], we are investigating PLLA that is quenched below T_g_ to create a largely amorphous preformed tube and heated to a temperature that provides enough mobility for crystallization to occur. Under these conditions, lamellae are slow to form [35]. Indeed, under most of the conditions we examined, negligible evidence of lamellar structure was observed (SAXS peaks associated with lamellar stacks were weak or absent), which may explain the lack of NHSK. Alternatively, the absence of NHSK might be a consequence of the unusual mutual orthogonality of the nanotube axis and the stretch direction in our experiments. Previous FIC studies of polymer nanocomposites examined flows that orient nanotubes and polymer in approximately the same direction, such that both the polymer shish and nanotubes support nucleation of crystals with the same c-axis orientation [40,41,43,45]. Here, the stretching direction, which is the preferred direction for the orientation of the PLLA crystals c-axis, and the direction along which the axes of the nanotubes align remain orthogonal throughout tube expansion, which could hinder NHSK formation. Instead, the PLLA crystals form with c-axis along the stretching direction (circumferential direction of the tube, giving (110)/(200) diffraction peaks along z in Figure 5b), just as they do in the absence of WS**_2_**NTs (Figure 4b) [42].

PLLA-based bioresorbable devices are moving forward in “below the knee” applications [70] while their application to coronary heart disease remains on hold. Three unmet needs in PLLA devices are cited as ongoing obstacles: “strut thickness, radiopacity, and deliverability” [71]. The present study is a step toward preclinical studies of PLLA/WS_2_NT devices that may ultimately yield a stronger material than neat PLLA and enable thinner struts, which could improve deliverability (ease of navigating the device through blood vessels to a lesion). The inclusion of a heavy element (tungsten) in the nanotubes could also improve radiopacity, which is needed to provide surgeons with real-time X-ray imaging to deliver the stent. While the present study does not examine the effects of WS**_2_**NTs on the solid-state properties of PLLA-based bioresorbable vascular scaffolds (BVS), the significant effect of WS**_2_**NTs on the process-induced microstructure, particularly the influence of one process step on the next—here, the influence of extrusion on tube expansion— is relevant to understanding and controlling the mechanical strength achieved in later manufacturing steps, especially during the crimping step [19]. Future research is needed to compare neat PLLA and PLLA/WS**_2_**NT with respect to the mechanical properties of expanded tubes, laser-cutting and, especially, structure development during crimping.

## 5. Conclusions

Comparison of neat PLLA and PLLA/WS_2_NT nanocomposite under systematically varied conditions relevant to production of bioresorbable vascular scaffolds (BVS) revealed an intriguing combination of ways in which PLLA and WS_2_NTs strongly interact (dispersion, plasticization, and nucleation) and ways in which they act independently (uncoupled orientations of nanotubes and PLLA crystals). This study relied on both (a) matched sets of extruded tubes of PLLA and PLLA/WS_2_NT and (b) development of a custom rig to control tube expansion and allow in situ acquisition of small-angle and wide-angle X-ray scattering (SAXS and WAXS) at a synchrotron source. We found that the nanotubes were strongly oriented along the axial direction in the extruded tube, a sign of good dispersion in the PLLA during extrusion. During tube expansion, however, the nanotubes only slightly reoriented despite the large circumferential strain imposed by the process. Although acting as nucleating agents, the nanotubes did not observably perturb the orientation of PLLA crystals; rather, in PLLA/WS_2_NT tubes, the orientation distribution of PLLA crystallization matched that of the neat polymer. Reminiscent of the interactions among sequential processing steps of BVS, we discovered that the dispersion and alignment of WS_2_NTs during the extrusion step influenced these examples of structure development in the subsequent tube expansion process.

Mechanical strength and radiopacity are important design goals for reviving clinical trials of PLLA-based BVS to treat coronary heart disease. Both needs might be addressed using WS_2_NTs. This fundamental study of microstructure development provides a foundation for future studies of the influence of WS_2_NTs on the strength of PLLA/WS_2_NT nanocomposites and their ability to achieve these design goals and advance the development of PLLA-based BVS. Based on the surprising effects we have found so far, further discoveries almost certainly lie ahead in studies of the effects of BVS manufacturing steps (tube expansion, laser cutting and crimping) on the mechanical properties of PLLA/WS_2_NT nanocomposite tubes.

## Data Availability

Not applicable.

## Supplementary Materials

The following are available online at https://www.mdpi.com/article/10.3390/polym13111764/s1, Figure S1. (a) TEM and (b) SEM images of WS_2_NTs before melt-mixing with the polymer. Figure S2. Alignment of the rig with the incident X-ray beam and detector system (only the WAXS detector is visible in the picture (sample-detector distance ~20 cm) while the SAXS detector is located at about 8.5 m from the sample. The polymer tube, molds and IR lamps are placed with their axis (z-axis in the manuscript) parallel to the black arrow. Figure S3. Block diagram of the implemented control loop showing schematically the sensors, actuators, the interfaces between sensors and system, and the software architecture of the control box. Figure S4. Temperature calibration curves for (a) neat PLLA and (b) PLLA/WS_2_NT tubes: temperature recorded by the IR sensor (T_IR_), by a thermocouple inserted inside the mold but outside the tube (T_mold_) and a thermocouple inserted inside the tube (T_tube_). Figure S5. Temperature calibration during the heating phase for neat PLLA (yellow trace) and PLLA/WS_2_NT (grey trace) tubes: reading from the IR sensor (T_IR_), a thermocouple inserted inside the mold but outside the tube (T_mold_) and a thermocouple inserted inside the tube (T_tube_). Figure 6. Diagram (not to scale) of tube cross section viewed from above (along the z-axis of the tube). On the left, the tube is well centered in the mold and the X-rays pass through the full thickness of the tube. On the right, the tube is shown to have moved off center in the mold, which can occur due to the effects of pressure and temperature during expansion. As a result, the X-rays pass through a different, possibly thinner, section of the tube. The material thickness diffracting the X-rays corresponds to the red dots and varies with the tube’s spatial position. Figure S7. Plots of the strain of the OD (blue solid line) and ID (blue dashed line) alongside the temperature ramp (orange line) are presented for the expansion of neat PLLA (top) and PLLA/WS_2_NT (bottom) tubes (T_ann_ = 70 °C) for T_act_ = 40, 50 and 60 °C. The gaps in the data points marked by vertical black dashes indicate time points at which the reflection of the heating lamps on the mold obscured measurement of the tube diameter. For the PLLA/WS_2_NT (T_act_ = 40 °C), the inception point of expansion was identified at t ~38 s, although the IR lamps’ reflection on the mold prevented a clear measurement of the OD. Figure S8. Quantitative characteristics of 1D WAXS profiles for expansion performed at T_ann_ = 70 °C with the pressure activated at T_act_ = 40 (blue), 50 (orange), and 60 °C (yellow) for neat PLLA (left) and PLLA/WS_2_NT (right). (a) intensity of the PLLA (110)/(200) peak (plain line) and amorphous halo (dotted line) masks; (b) FWHM (I vs ϕ) of the PLLA (110)/(200) peak. For PLLA/WS_2_NT tubes: (c) FWHM (I vs ϕ) of WS_2_ (002) peak; and (d) FWHM (I vs ϕ) of SAXS peak, averaged every 8 and 64 frames to reduce the noise. Initial data points are missing for T_act_ = 60 °C PLLA/WS_2_NT because the tube was not aligned within the X-ray path. Figure S9. Plots of the strain of the OD (blue solid line), ID (blue dashed line) measured on the camera images alongside the temperature ramp (orange line) are presented for the expansion of neat PLLA samples (T_act_ = 40 °C T_ann_ = 80 °C) at the pressure values of of 7, 8 and 9 bar. Figure S10. (a) Camera images, in situ (b) WAXS and (c) SAXS data acquired from a neat PLLA sample at two different times (t = −10 and 41 s) before the start of expansion. Figure S11. (a) Transmitted light microscopy image of the outside surface of the neat PLLA tube after full expansion. The marks visible in the machine direction (z-direction) are weld lines created around the die during the tube extrusion. (b) Transmitted light microscopy image and (c) SEM image of the inside surface of the neat PLLA tube after full expansion. The z-oriented features are attributed to the strong deformation along the circumferential direction of the tube, which could be up to three times higher along the inner surface of the tube than along the outer. Figure S12. (a) Intensities of the (110)/(200) PLLA peak normalized by the thickness of tube (plain line) and halo (dotted line) masks (shown in Figure 4 and Figure 5) and (b) FWHM of the PLLA (110)/(200) peak for neat PLLA (green) and PLLA/WS_2_NT (purple) tubes during the heating and annealing steps. The temperatures shown are the annealing temperatures (T_act_ = 40 °C for all samples). Figure S13. (a) Setup for the expansion in water bath; the PLLA tube is stretched at the extremities and connected to the rig pressure inlet. When the rig is inserted in the water bath, mechanical tension is applied to the tube and the pressure valve is opened. (b) Expanded tubes and relative OD (Ø) obtained after expansion in a water bath at 60 °C (left: PLLA, right: PLLA/WS_2_NT).
