# Supplementary material for: Interaction of Poly L-Lactide and Tungsten Disulfide Nanotubes Studied by In Situ X-ray Scattering during Expansion of PLLA/WS2NT Nanocomposite Tubes
Source: Polymers (Basel). 2021 May 27;13(11):1764. doi: 10.3390/polym13111764 (PMC8198810; doi:10.3390/polym13111764)
Supplement: Supplementary file 1 [file polymers-13-01764-s001.zip › polymers-1212282-supplementary.pdf]

polymers

MDPI

Supplementary Material

Interaction of Poly L-Lactide and Tungsten Disulfide Nanotubes Studied by in Situ X-Ray Scattering during Expansion of PLLA/WS_2NT Nanocomposite Tubes

Lison Rocher [1], Andrew S. Ylitalo [2], Tiziana Di Luccio [2], Riccardo Miscioscia [3], Giovanni De Filippo [4], Giuseppe Pandolfi [3], Fulvia Villani [3], Alla Zak [5], Gary H. Menary [1], Alex B. Lennon [1] and Julia A. Kornfield [2,*]

[1] School of Mechanical and Aerospace Engineering, Queen's University Belfast, Belfast BT9 5AH, UK; l.rocher@qub.ac.uk (L.R.); G.Menary@qub.ac.uk (G.H.M.); a.lennon@qub.ac.uk (A.B.L.)
[2] Division of Chemistry and Chemical Engineering, California Institute of Technology, Pasadena, CA 91125, USA; aylitalo@caltech.edu (A.S.Y.); tidilu@caltech.edu (T.D.L.)
[3] Division of Sustainable Materials, ENEA, Centro Ricerche Portici, 80055 Portici, Italy; riccardo.miscioscia@enea.it (R.M.); giuseppe.pandolfi@enea.it (G.P.); fulvia.villani@enea.it (F.V.)
[4] Division of Photovoltaics and Smart Networks, ENEA, Centro Ricerche Portici, 80055 Portici, Italy; giovanni.defilippo@enea.it
[5] Faculty of Sciences, Holon Institute of Technology, Holon 5810201, Israel; alzak@hit.ac.il
[*] Correspondence: jak@cheme.caltech.edu; Tel.: +1-626-395-4138

Citation: Rocher, L.; Ylitalo, A.S.; Luccio, T.D.; Miscioscia, R.; De Filippo, G.; Pandolfi, G.; Villani, F.; Zak, A.; Menary, G.H.; Lennon, A.B.; et al. Interaction of Poly L-Lactide and Tungsten Disulfide Nanotubes Studied by in Situ X-ray Scattering during Expansion of PLLA/WS_2NT Nanocomposite Tubes. *Polymers* **2021**, *13*, 1764. https://doi.org/10.3390/polym13111764

Academic Editor: Young-Sam Cho

Received: 21 April 2021
Accepted: 24 May 2021
Published: 27 May 2021

Publisher's Note: MDPI stays neutral with regard to jurisdictional claims in published maps and institutional affiliations.

Copyright: © 2021 by the authors. Licensee MDPI, Basel, Switzerland. This article is an open access article distributed under the terms and conditions of the Creative Commons Attribution (CC BY) license (http://creativecommons.org/licenses/by/4.0/).

Microscopy Images of WS_2NTs

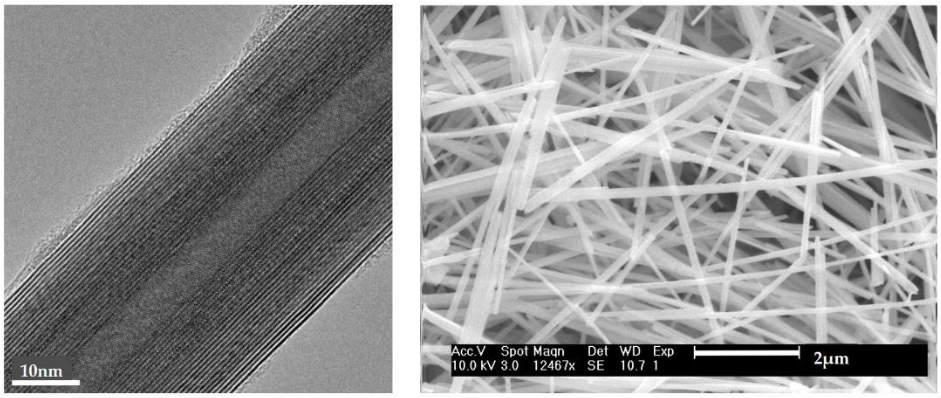

(**a**) (**b**)

Figure S1. (**a**) TEM and (**b**) SEM images of WS_2NTs before melt-mixing with the polymer.

Implementation of the Tube Expansion Instrument at the X-Ray 5-ID-D Beamline at the Advanced Photon Source of Argonne National Laboratories

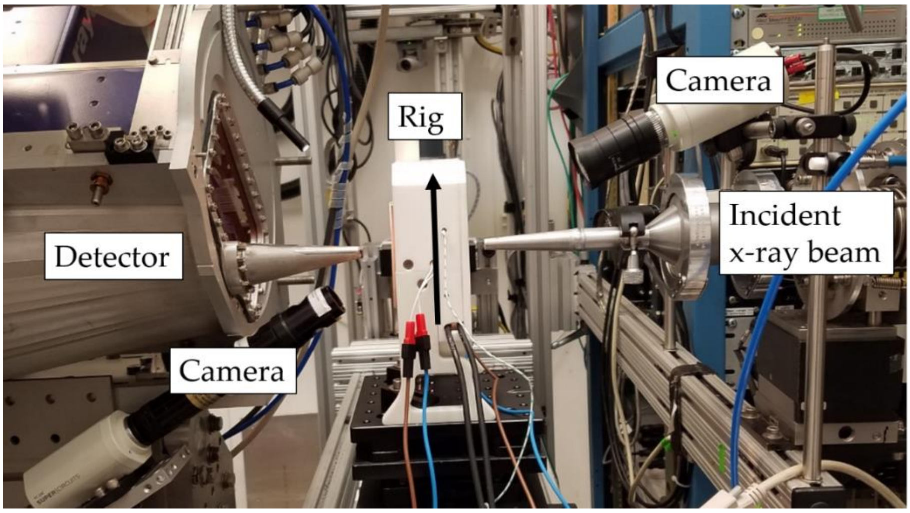

Figure S2. Alignment of the rig with the incident X-ray beam and detector system (only the WAXS detector is visible in the picture (sample - detector distance ~20 cm) while the SAXS detector is located at about 8.5 m from the sample. The polymer tube, molds and IR lamps are placed with their axis (z-axis in the manuscript) parallel to the black arrow.

Additional Details on the Rig Mechanical Assembly and Control Box

The mechanical assembly is held in place by a platform to fix it to an X-Y translation stage present in the beamline laboratory that we used to align the sample to the X-ray path. The platform bears two brackets that support the PTFE body of the assembly. In its framework, are two lamp holders with reflectors applied to concentrate the infrared light onto the mold and the polymer tube. The tube is inserted vertically from the top of the assembly. A screw is used to lock the weight holder to the sealed tube end, to which weights can be applied to cause additional axial deformation. In the bottom part of the mechanical assembly, a Hall sensor detects the magnetic field generated by a neodymium permanent magnet facing it and fixed to the weight's holder. The infrared (IR) thermometer faces the mold and the tube in the bottom part of the enclosure and its body is shielded from infrared light coming from the lamps by the PTFE housing.

The control box is based on an Arduino Mega 2560 board (microcontroller unit, MCU), Italy that operates at a 16 MHz clock speed to acquire data from the sensors and to drive the actuators (Figure S3). The MCU drives a dual MOSFET driver stage (PWM MOSFET driver) which supplies electrical power to the two heaters (two 500 W Philips T3 halogen lamps, as described in the paper of Ramachandran by al. [1]). The lamp driver is a dual common-source switching circuit based on logic-level Infineon IRL540N 100V Single N-Channel power MOSFETs. If compared to the previous version of the control box in Ramachandran et al. [1], no relay has been applied to shut off the lamps because the same effect is obtained by zeroing the duty-cycle of the Pulse Width Modulated (PWM) input to the MOSFET gates. A software-defined PID (Proportional–Integral–Derivative) controller routine is coded into the firmware of the control box to assure proper closed-loop operation of the temperature control feedback (block: "PID controller") and its coefficients (P, I, D) are determined experimentally for stable and accurate regulation. A reference generator computes the temperature setpoint as an input to the PID controller according to the user-supplied recipe. The software-based reference generator can output a temperature ramp with user-definable temperature-rate to heat the tube faster or slower.

A normally closed pressure electrovalve (here, 3/2 NC SMC VDW350-5G-4-02F-Q, 1 MPa max, 24VDC) is controlled by the MCU through an opto-isolated 5 V relay that triggers the expansion process when programmable process conditions are reached.

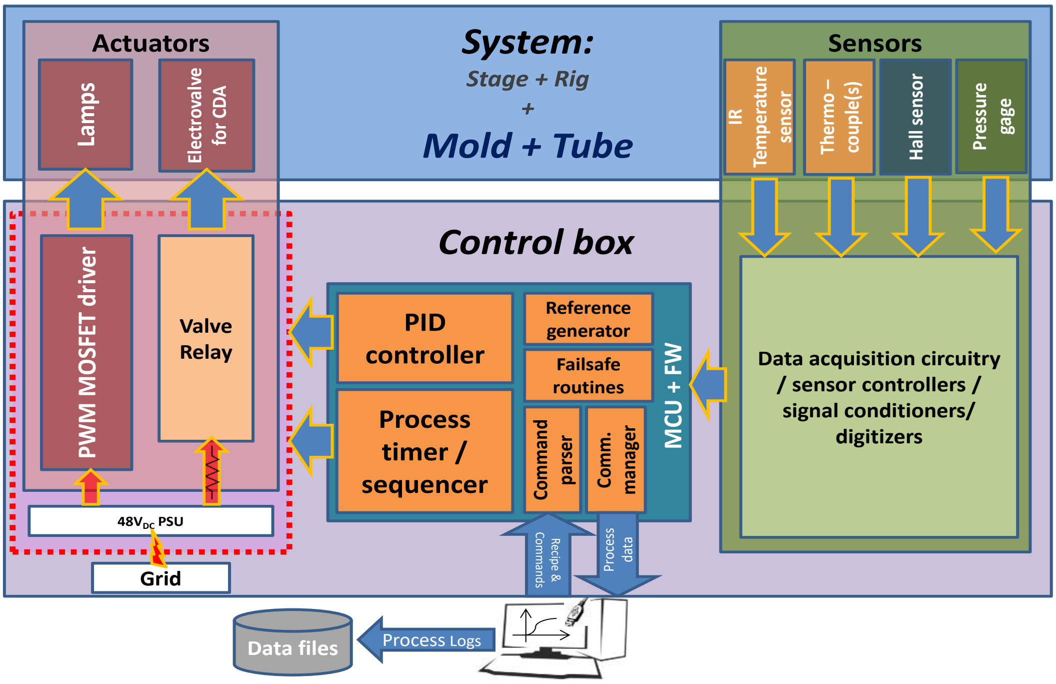

Figure S3. Block diagram of the implemented control loop showing schematically the sensors, actuators, the interfaces between sensors and system, and the software architecture of the control box.

The control-logic of the instrument gathers data from an IR sensor (MELEXIS MLX90614ESF-BCC Integrated Infrared Thermometer 3V, single zone, standard accuracy, gradient compensated, 35° viewing angle) which is interfaced to the MCU through an I2C-compatible interface and inputs the measured temperature to the PID controller for temperature feedback control. In this version of the firmware, inside the declaration of the tube expansion recipe, the operator can choose to utilize the temperature reading from the IR sensor or from a standard K-type thermocouple to perform the temperature control. The thermocouple is connected to the MCU through a Maxim MAX6675 cold-junction compensated thermocouple controller (OLIMEX MOD-TC-MK2 Max 6675 thermocouple controller board, similar to that described in the article from Ramachandran et al. [1]) wired through an SPI (Serial Peripheral Interface) interface to the Arduino board. Data is acquired from the sensors by the MCU at defined and equally spaced time intervals (sampling period). For each sampling period, the MCU also acquires and logs (via the USB interface) the digitized values from a magnetic field Hall sensor and from a pressure gauge attached to the pressure pipe at the to the top part of the tube. The magnetic field sensor is the Honeywell SS49E linear Hall-effect sensor IC (Ratiometric Board Mount Hall Effect/Magnetic Sensor) and is powered by the reference voltage from the MCU and its output acquired through an analog input available on the Arduino board. The SS49E is faced to a permanent magnet that is fixed to the support for the elongation weights. Then, the magnetic field output is digitized by the MCU through its integrated Analog to Digital Converter (ADC) with a resolution of 10 bits in the range 0-5 V. In this configuration, the sensor can detect magnetic fields between −1000 G and +1000 G with a resolution of less than 2 G for a variation in the less significant bit. The operator can manually set the minimum variation of the magnetic field (expressed in arbitrary units) for the proximity sensor that triggers the opening of the pressure valve in the case when the control box is using the corresponding failsafe behavior.

The control box can perform three types of "failsafe" behaviors capable of changing the process: "type 0" (or "f0"), "type 1" ("f1") and "type 2" ("f2"). When operating in f0 mode, the value of the magnetic sensor is logged but ignored from the failsafe routines and no variation in the user-defined recipe is applied when the movement starts. When the "f1" failsafe mode is activated in the processing recipe, if the control box detects a

sufficient movement of the weight holder (higher than the threshold defined by the user), and the reference generator is performing a heating ramp, the pressure valve is immediately opened, the ramp is stopped and the temperature is stabilized at the current value. In this case, the temperature setpoint is fixed at the reference generator and then at the PID controller. When operated in f2 mode, during the heating stage, if the tube movement is detected above user-defined limits, the pressure valve is opened to perform the expansion, but the temperature ramp continues to run until the setpoint is reached. Upon reaching the setpoint, the control box switches to a constant temperature operation. This last type of failsafe mode can be utilized to fix the annealing temperature while adapting the expansion temperature to match the temperature at which the tube begins to elongate in that experiment.

Finally, a Java™-based desktop application acts as a terminal through which the operator communicates with the control box. In manual mode, the operator issues text commands to the control box. Otherwise, the operator may submit a full process recipe when performing repeated experiments. The same desktop application creates plots of the data and logs them to a text file.

Calibration

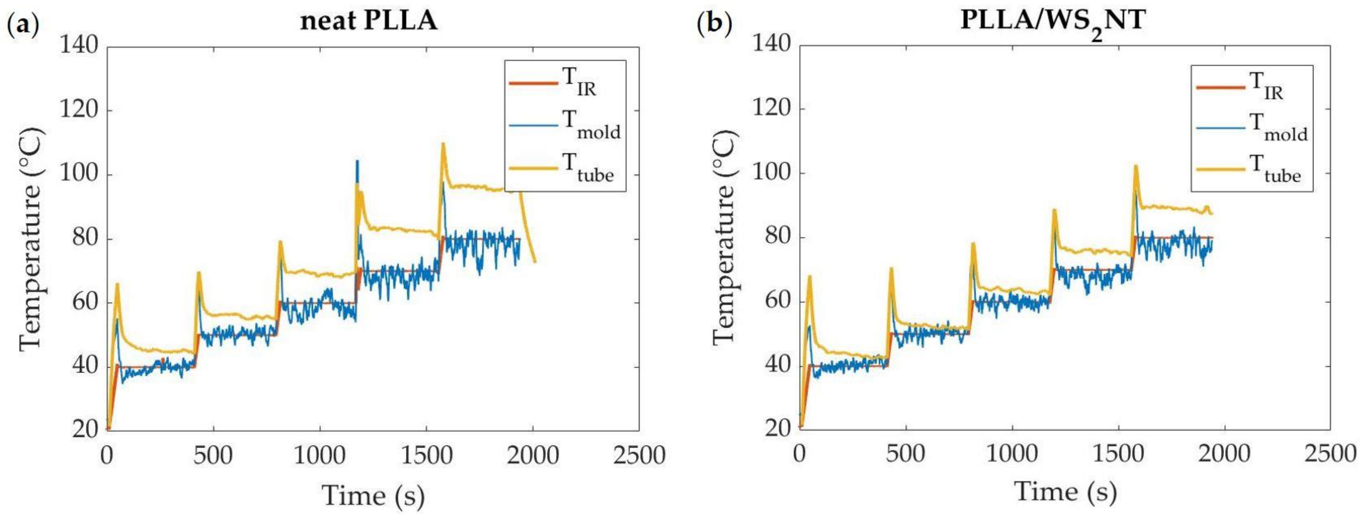

Figure S4. Temperature calibration curves for (**a**) neat PLLA and (**b**) PLLA/WS_2NT tubes: temperature recorded by the IR sensor (T_{IR}), by a thermocouple inserted inside the mold but outside the tube (T_{mold}) and a thermocouple inserted inside the tube (T_{tube}).

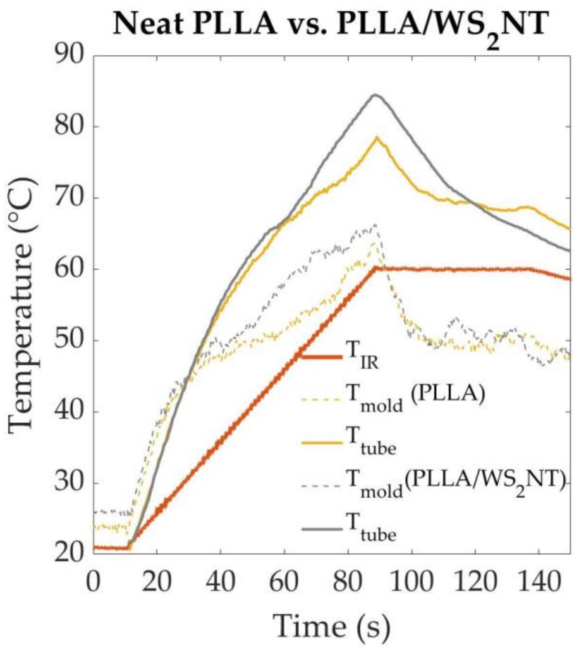

Figure S5. Temperature calibration during the heating phase for neat PLLA (yellow trace) and PLLA/WS_2NT (grey trace) tubes: reading from the IR sensor (T_{IR}), a thermocouple inserted inside the mold but outside the tube (T_{mold}) and a thermocouple inserted inside the tube (T_{tube}).

Disparities between neat PLLA and PLLA/WS_2NT tubes could be attributed to the difference in IR absorption of the two materials (WS_2NTs are expected to increase IR absorption due to their darker color).

Misalignment of the Tube with the X-Ray Path

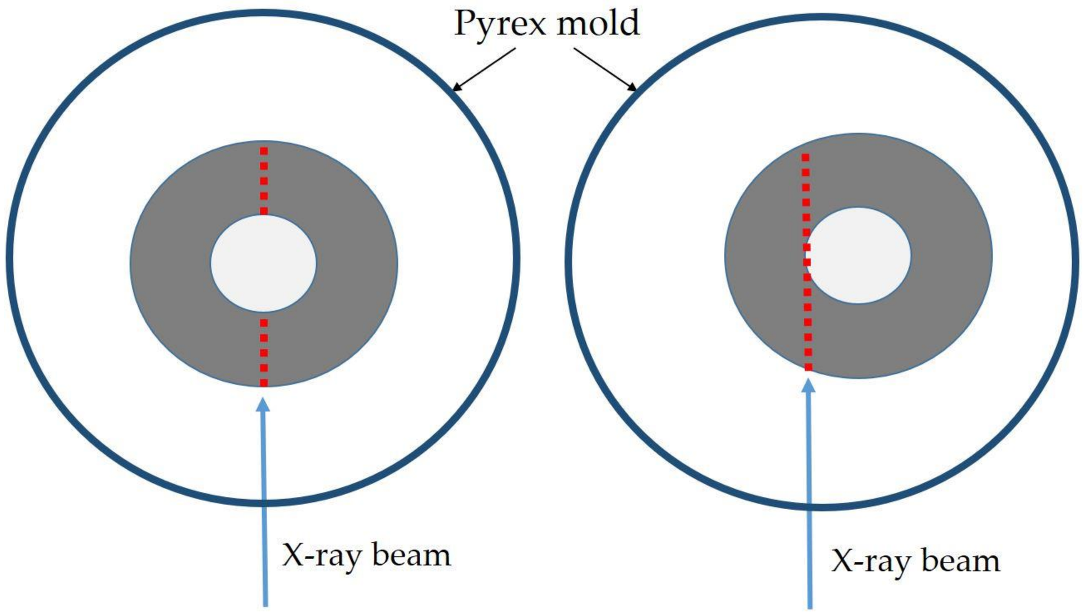

Figure 6. Diagram (not to scale) of tube cross section viewed from above (along the z-axis of the tube). On the left, the tube is well centered in the mold and the X-rays pass through the full thickness of the tube. On the right, the tube is shown to have moved off center in the mold, which can occur due to the effects of pressure and temperature during expansion. As a result, the X-rays pass through a different, possibly thinner, section of the tube. The material thickness diffracting the X-rays corresponds to the red dots and varies with the tube's spatial position.

Strain Field during Expansion at Different Pressure Activation Temperatures

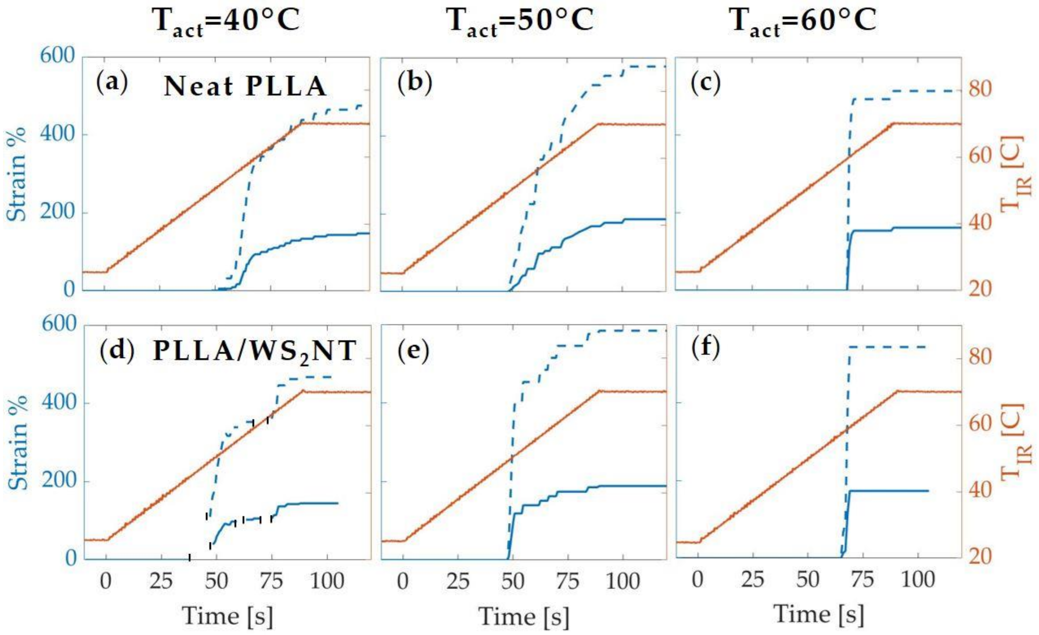

Figure S7. Plots of the strain of the OD (blue solid line) and ID (blue dashed line) alongside the temperature ramp (orange line) are presented for the expansion of neat PLLA (**a**,**b**,**c**) and PLLA/WS_2NT (**d**,**e**,**f**) tubes (T_{ann} = 70 °C) for T_{act} = 40, 50 and 60 °C. The gaps in the data points marked by vertical black dashes indicate time points at which the reflection of the heating lamps on the mold obscured measurement of the tube diameter. For the PLLA/WS_2NT (T_{act} = 40 °C), the inception point of expansion was identified at t ~38 s, although the IR lamps' reflection on the mold prevented a clear measurement of the OD.

To study the effect of pressure activation temperature, a full set of samples was only collected for T_{ann} = 70 °C due to the limited time at the beamline. When the pressure was activated at 40 °C (Figure S7-left), the tube had not yet fully softened, so the tubes did not blow until they softened enough under the heat that they could no longer withstand the pressure. Blowing began just above 40 °C for the PLLA/WS_2NT tube, but did not begin until the temperature reached ~50 °C for the neat PLLA tube. For both tubes, the evolution of strain passed through the two phases described in the main text: phase I of the expansion occurred at a strain rate around ~0.1 s^{-1}, followed by a decrease in the strain rate to ~0.04 s^{-1} during phase II until the end of the expansion. When the pressure was activated at 50 °C (Figure S7-middle), the neat PLLA tube underwent two-phase expansion while the PLLA/WS_2NT tube expanded immediately at a much faster strain rate (~1 s^{-1}) before slowing down for the rest of the expansion. When the pressure was activated at 60 °C (Figure S7-right), both samples expanded at a strain rate around ~1 s^{-1}, but did so too quickly to distinguish two-phase expansion.

Characteristics of the Waxs 1D Profiles during Expansion at Different Pressure Activation Temperatures

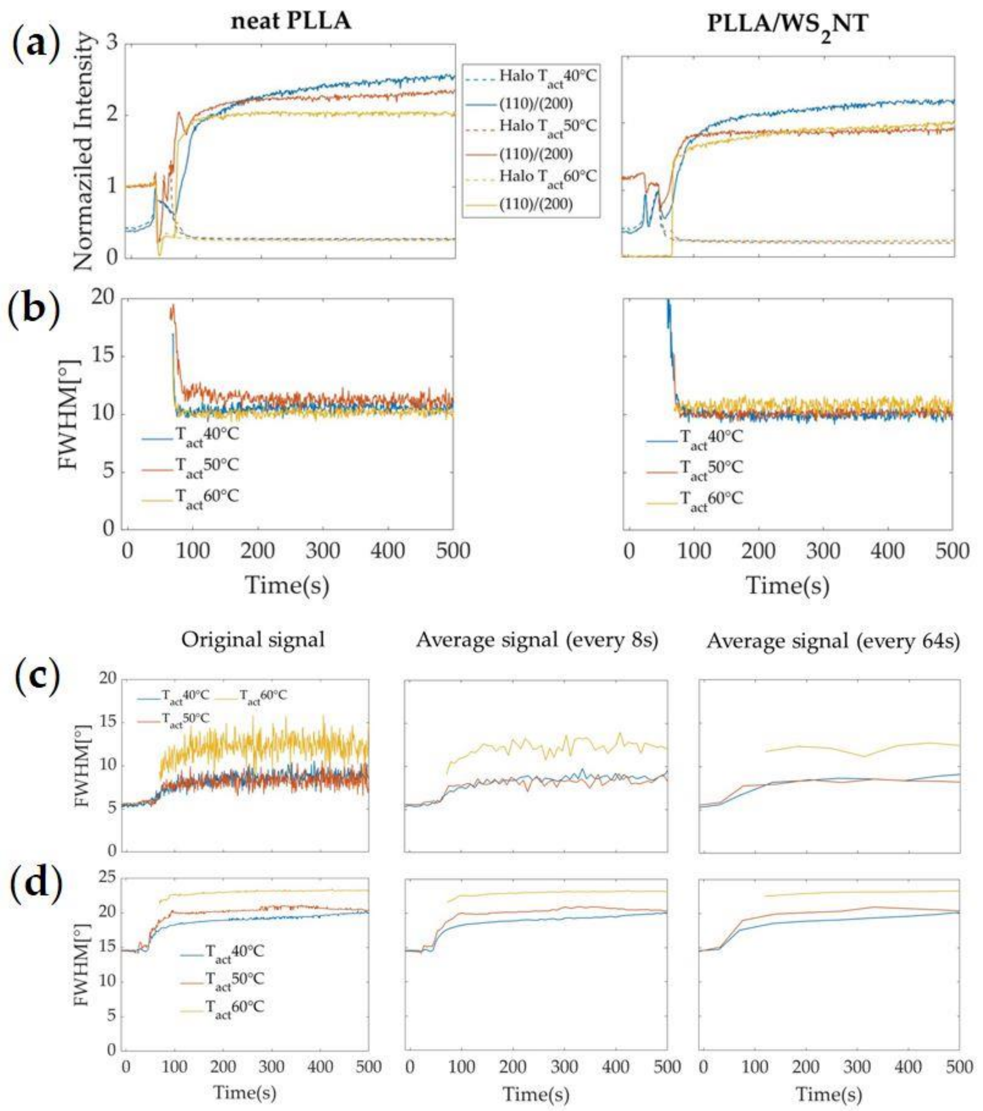

Figure S8. Quantitative characteristics of 1D WAXS profiles for expansion performed at T_{ann} = 70 °C with the pressure activated at T_{act} = 40 (blue), 50 (orange), and 60 °C (yellow) for neat PLLA (left) and PLLA/WS_2NT (right). (**a**) intensity of the PLLA (110)/(200) peak (plain line) and amorphous halo (dotted line) masks; (**b**) FWHM (I vs ϕ) of the PLLA (110)/(200) peak. For PLLA/WS_2NT tubes: (**c**) FWHM (I vs ϕ) of WS_2 (002) peak; and (**d**) FWHM (I vs ϕ) of SAXS peak, avaeraged avery 8 and 64 frames to reduce the noise. Initial data points are missing for T_{act} = 60 °C PLLA/WS_2NT because the tube was not aligned within the X-ray path.

Here, we explore the evolution of the microstructure of the samples presented in Figure S7. At T_{act} = 60 °C, the rapid deformation (strain rate ~1 s^{-1}), triggered by the pressure activation, is accompanied by an immediate increase in the intensity of the (110)/(200) θ-oriented peak for both neat PLLA and PLLA/WS_2NT tubes. Once the expansion complete, almost no further increase is observed for the rest of the experiment (Figure S8a). The azimuthal width of the (110)/(200) peak after expansion does not seem to be influenced by the change of strain rate (FWHM ~10 ° for all pressure activated temperatures on Figure S8b). Final values of FWHM of the SAXS and WAXS peaks for T_{act} = 60 °C seem higher compared to T_{act} = 40 and 50 °C (Figure S8c,d) but initial values are missing to be able to compare the increase of FWHM.

Strain Field during Expansion at Different Pressures

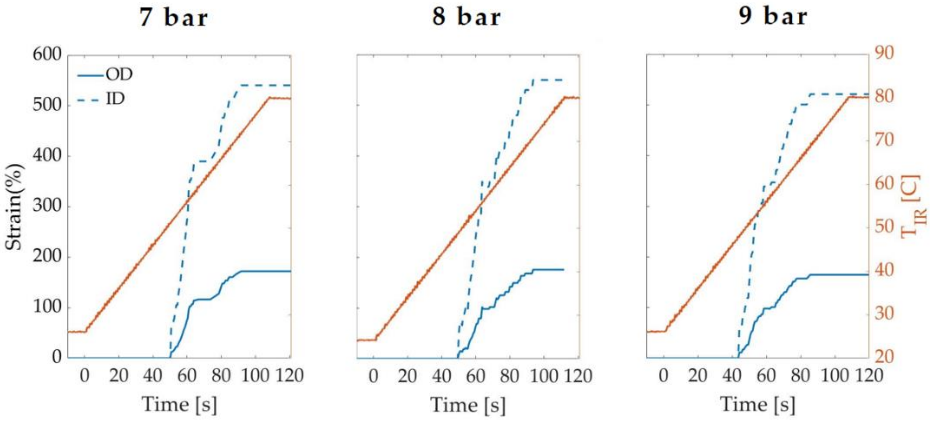

Figure S9. Plots of the strain of the OD (blue solid line), ID (blue dashed line) measured on the camera images alongside the temperature ramp (orange line) are presented for the expansion of neat PLLA samples (T_{act} = 40 °C T_{ann} = 80 °C) at the pressure values of of 7, 8 and 9 bar.

We also tested the effect of the pressure on tube expansion. Because blowing was not satisfactory below 7 bar and the risk of bursting the tube became too great above 9 bar, we present strain results for pressures of 7, 8, and 9 bar (at T_{act} = 40 °C and T_{ann} = 80 °C) in Figure S9. Due to the small differences in strain and strain rate at the pressures considered, the pressure was fixed between 7 and 8 bar for the remaining experiments.

Streak on SAXS 2D Pattern

Prior to and during the heating phase of the expansion experiments, sharp and transient streaks, parallel to tube axial direction, can appear on the SAXS diffraction pattern (Figure S10c). On the top image, the tube has shifted slightly to the right side of the mold, such that the X-ray beam does not pass directly through its center. Rather, the beam passes through the sharp interface along the left external wall on the tube, causing a sharp peak with high intensity to emerge on the radially averaged intensity plot (blue trace). On the bottom image, the tube has returned to the center of the mold, and the beam now passes through the center of the tube. As a result, a full, brighter amorphous hollow can be seen in the WAXS pattern and the streak in the SAXS pattern has almost disappeared. However, a remaining z-oriented peak is visible in the radially averaged intensity plot, which suggests the presence of an oriented scattering pattern parallel to the z-direction of the tube. This could be attributed to shish precursors induced during the extrusion process, but the absence of oriented crystals in the WAXS pattern at the beginning of extrusion does not support this hypothesis.

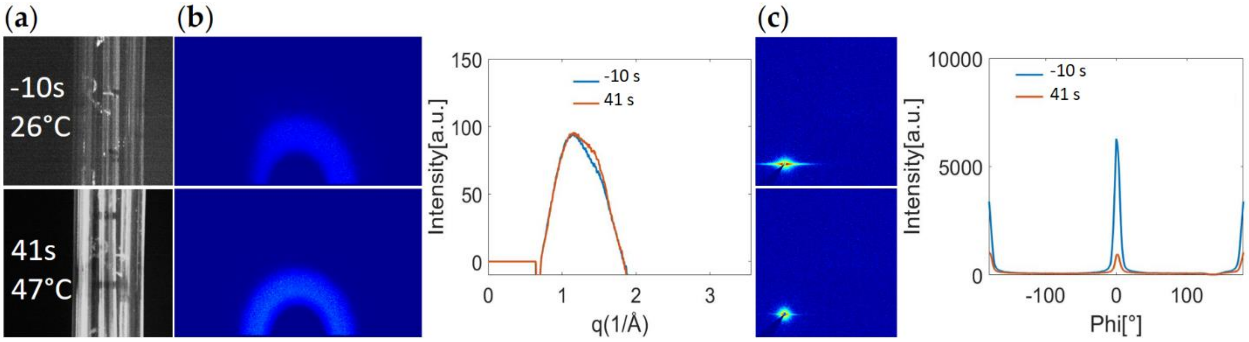

Figure S10. (**a**) Camera images, in situ (**b**) WAXS and (**c**) SAXS data acquired from a neat PLLA sample at two different times (t = −10 and 41 s) before the start of expansion.

Microscope Images of Expanded Neat PLLA Tubes

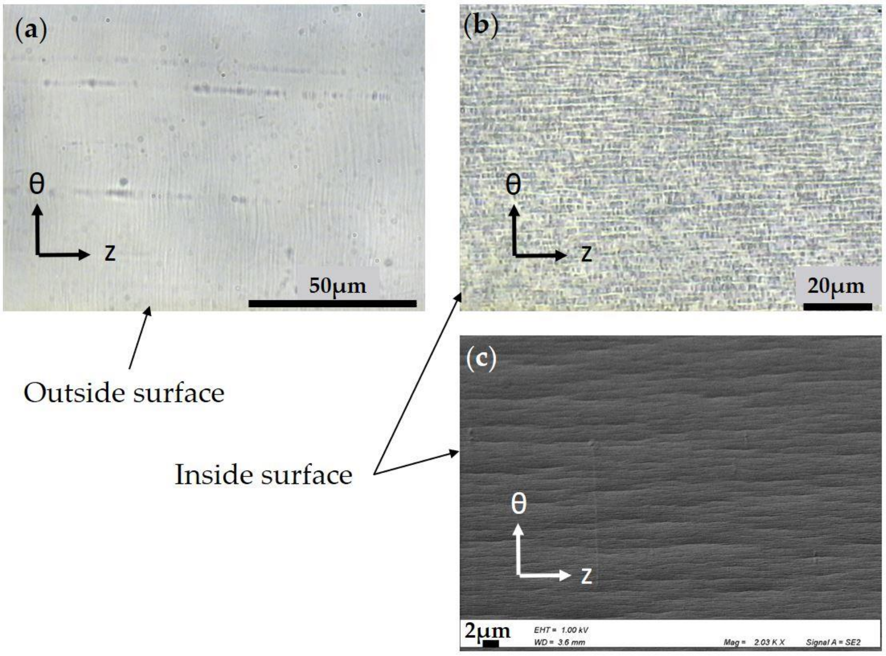

Figure S11. (**a**) Transmitted light microscopy image of the outside surface of the neat PLLA tube after full expansion. The marks visible in the machine direction (z-direction) are weld lines created around the die during the tube extrusion. (**b**) Transmitted light microscopy image and (**c**) SEM image of the inside surface of the neat PLLA tube after full expansion. The z-oriented features are attributed to the strong deformation along the circumferential direction of the tube, which could be up to three times higher along the inner surface of the tube than along the outer.

The strong horizontal streak in the SAXS pattern of the nanocomposite (Figure 5c) is consistent with the presence of long, slender particles having their long axis along the tube axis, which accords well with the alignment of WS_2NTs observed in WAXS (Figure 5b). The much weaker horizontal streak observed in the neat PLLA (Figure 4c) is harder to explain. Although we do not know the origin of this horizontal peak, we note that it might be related to previously reported formation of nano-cavities and micro-voids [2–4], which is consistent with the concurrent whitening of the neat PLLA material (video frames in Figure 4a). To evaluate this hypothesis, we observed tubes after expansion with transmitted light and scanning electron microscopy to look for linear features oriented perpendicular to the deformation direction. Indeed, elongated structures were seen on the inner surface of the tube (Figure S11b,c), but were not seen on the outside surface (Figure S11a). These features are commonly observed in stretched polymers and can indicate incipient micro-voids, which are more likely to form on the inner surface due to the high ID strain (>400%) with respect to the OD (160%). These long, slender features oriented along the z-direction could give rise to a SAXS streak in the meridional direction. The relatively weak intensity of the SAXS streak could be attributed to the limited presence of these features to the inner surface of the tube.

(110)/(200) PLLA Peak Intensity and FWHM during Expansion and Annealing

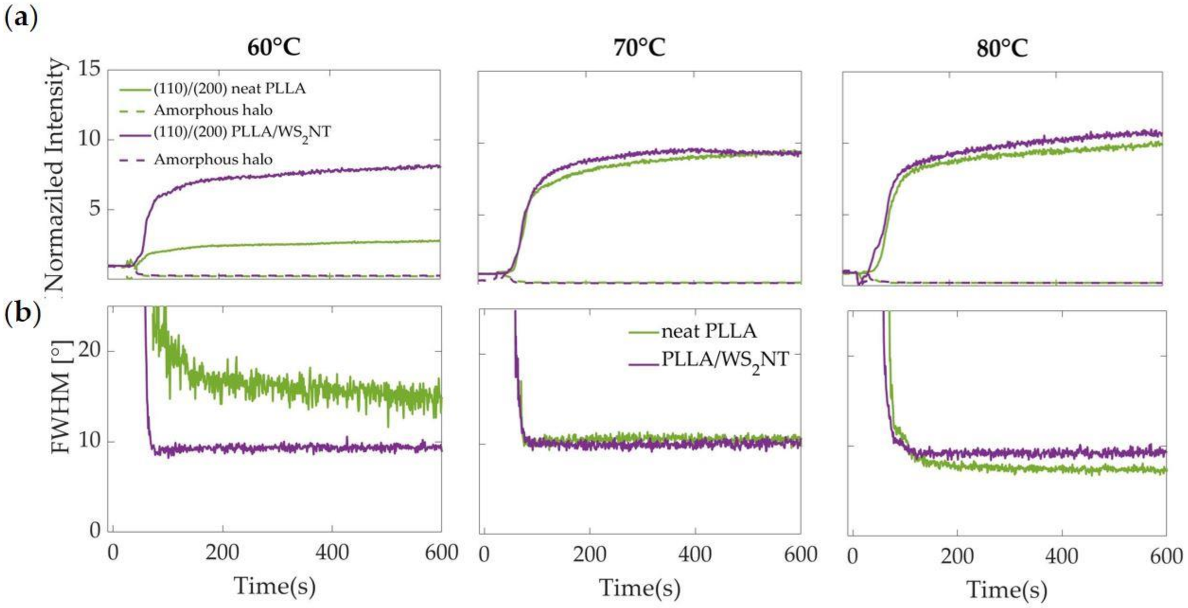

Figure S12. (**a**) Intensities of the (110)/(200) PLLA peak normalized by the thickness of tube (plain line) and halo (dotted line) masks (shown in Figures 4 and 5) and (**b**) FWHM of the PLLA (110)/(200) peak for neat PLLA (green) and PLLA/WS_2NT (purple) tubes during the heating and annealing steps. The temperatures shown are the annealing temperatures (T_{act} = 40 °C for all samples).

Expansion in Water Bath

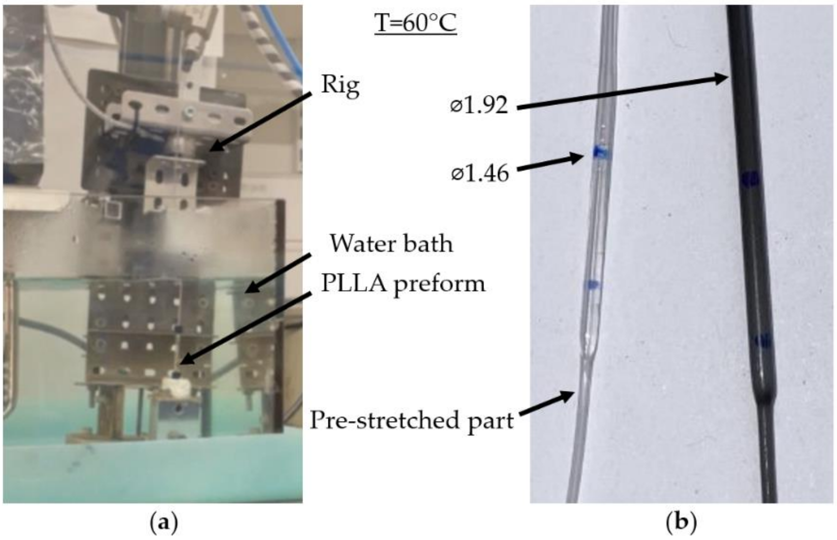

Figure S13. (**a**) Setup for the expansion in water bath; the PLLA tube is stretched at the extremities and connected to the rig pressure inlet. When the rig is inserted in the water bath, mechanical tension is applied to the tube and the pressure valve is opened. (**b**) Expanded tubes and relative OD (∅) obtained after expansion in a water bath at 60 °C (left: PLLA, right: PLLA/WS_2NT).

Some additional expansion experiments were performed using another rig developed at Queen's University Belfast (Figure S13a) to investigate the effect of WS_2NTs on the minimum temperature required to blow a PLLA tube. The tubes were heated using a water bath to remove the effect of differences in infrared (IR) absorption between the materials. Before the experiment, the extremities of the tubes were pre-stretched and connected to the pressure inlet at one end and clamped to the rig at the other end. Then, the

rig was immersed in the water bath (already set to the targeted temperature), a tension was applied to the tube, and the pressure valve was opened to release compressed gas that drove the expansion. During the first blowing experiment, conducted at T = 70 °C, both tubes were able to blow and reached the same diameter at the end of the experiment. Lower temperatures were tested until a difference in blowing was observed between the neat PLLA and PLLA/WS_2NT tubes. At T = 60 °C, the comparison of the tubes OD indicates a larger deformation for the PLLA/WS_2NT compared to neat PLLA, which indicates a plasticizing effect of the nanotubes (Figure S13b).

References

1. Ramachandran, K.; Miscioscia, R.; De Filippo, G.; Pandolfi, G.; Di Luccio, T.; Kornfield, J.A. Tube Expansion Deformation Enables In Situ Synchrotron X-ray Scattering Measurements during Extensional Flow-Induced Crystallization of Poly l-Lactide Near the Glass Transition. *Polymers* **2018**, *10*, 288, doi:10.3390/polym10030288.
2. Billimoria, K.; Heeley, E.L.; Parsons, N.; Figiel, Łukasz An investigation into the crystalline morphology transitions in poly-L-lactic acid (PLLA) under uniaxial deformation in the quasi-solid-state regime. *Eur. Polym. J.* **2018**, *101*, 127–139, doi:10.1016/j.eurpolymj.2018.01.031.
3. Lu, Y.; Men, Y. Cavitation-Induced Stress Whitening in Semi-Crystalline Polymers. *Macromol. Mater. Eng.* **2018**, *303*, 31, doi:10.1002/mame.201800203.
4. Renouf-Glauser, A.C.; Rose, J.; Farrar, D.F.; Cameron, R.E. The effect of crystallinity on the deformation mechanism and bulk mechanical properties of PLLA. *Biomaterials* **2005**, *26*, 5771–5782, doi:10.1016/j.biomaterials.2005.03.002.
